# The deletion of the arginine vasopressin 1a receptor impairs sexual and maternal behavior

**DOI:** 10.3389/fendo.2025.1649706

**Published:** 2025-09-17

**Authors:** D. Aspesi, E. Sambor, M. C. Stoehr, J. Taylor, Z. A. Grieb, K. L. Huhman, H. E. Albers

**Affiliations:** Neuroscience Institute, Georgia State University, Atlanta, GA, United States

**Keywords:** arginine-vasopressin, vasopressin receptor 1a, sexual behavior, maternal behavior, Syrian hamster

## Abstract

**Introduction:**

Arginine-vasopressin (AVP) and its V1a receptor (V1aR) within the social behavior neural network are critical modulators of social behavior across species. Using CRISPR-Cas9-mediated gene editing, we previously demonstrated that Syrian hamsters of both sexes with V1aR knockout (V1aRKO) exhibit elevated social communication (i.e., odor-induced flank marking) and aggression compared to wild-type (WT) littermates. While most AVP research has focused on males, V1aRs have also been implicated in female sexual and maternal behaviors. Here, we investigated the effects of V1aRKO on reproductive and maternal behavior in adult female hamsters.

**Methods:**

To assess mating behavior, a sexually experienced male hamster was introduced into the home cage of a virgin, estrus female, and their interactions were video-recorded for 30 minutes following the male's first ejaculation or until the female displayed aggression. To evaluate maternal care, home-cage behavior was video-recorded for 5 minutes on postpartum days (PPD) 0 to 3, across four timepoints per day (two times in the dark and two in the light phase), and pup-directed and non-directed behaviors were quantified.

**Results:**

V1aRKO and heterozygous (Het) females received significantly fewer and shorter-duration mounts from males than did WT females, suggesting reduced sexual receptivity or attractiveness. Other copulatory and social behaviors, including aggression, during the observation period were unaffected. In regard to maternal behavior, V1aRKO females exhibited reduced pup-directed behaviors relative to Het and WT dams, although time spent in the nest was comparable across groups. V1aRKO females also engaged in more non-maternal behaviors (e.g., self-grooming, digging, and burying) than did Het or WT dams.

**Conclusions:**

These findings support the hypothesis that V1aRs are essential for the robust expression of female sexual receptivity and maternal caregiving in Syrian hamsters. This work underscores the importance of studying the AVP system across sexes and species to fully understand its role in regulating complex social behaviors.

## Introduction

Successful reproduction is key to a species’ evolutionary success. To increase the survival chances of offspring, many species exhibit varying degrees of parental behavior. In approximately 90% of species, uniparental care predominates, with mothers providing the primary support to offspring (1, 2). In rodents, maternal care includes nutritional provisioning through lactation, along with a range of behaviors such as kyphosis/crouching to facilitate nursing and licking/grooming pups to aid in urination and defecation (2). Other maternal behaviors that dramatically increase the survival of the offspring include the defense of the pups against potential threats like an intruding conspecific (*maternal aggression*) and retrieving pups into the nest when they move away from it (*pup retrieval*) (3). These behaviors depend on coordinated neuroendocrine changes in the mother’s brain, with neuropeptides such as oxytocin (OT) and arginine-vasopressin (AVP) playing central roles (4). While oxytocin (OT) has been extensively studied in relation to parturition and maternal bonding, AVP is increasingly recognized as a critical modulator of maternal care, maternal aggression, and social memory (for a review see (5–7).

AVP is synthesized in the hypothalamus and acts centrally via V1a and V1b receptors (8). Among these, the V1a receptor (V1aR) has been repeatedly implicated in regulating social behaviors across species, particularly in males (9). In male rodents, AVP signaling via V1aR contributes to aggression, social recognition, and pair bonding (10–13). In female rats, evidence suggests that AVP may suppress lordosis (14) and interact with OT to shape sexual receptivity (15). While the effects of AVP on male sexual behavior appear limited, it appears to inhibit female lordosis in several species (16). The involvement of AVP in sexual behavior has been explored in several species. For instance, AVP modulates sexual vocalizations in male squirrel monkeys (17) and several aspects of copulatory behaviors in mice of both sexes (18). In Syrian hamsters (*Mesocricetus auratus*), AVP strongly influences aggression, flank marking, and other aspects of social communication (19, 20). Photoperiodic manipulations that reduce reproductive activity are also associated with changes in V1aR binding in brain regions regulating sociosexual behavior (21). In humans, AVP enhances recognition of sexual cues in males, suggesting a role in anticipatory rather than consummatory sexual behaviors (22). These findings highlight the complexity of V1aR contributions to sexual signaling and suggest that its role in female reproduction may be species- and context-dependent.

In addition to sociosexual and affiliative behaviors, AVP and V1aR has been linked to maternal behavior. Manipulations of V1aR in rats, mice, and voles have demonstrated its involvement in maternal responsiveness, pup-directed care, and maternal aggression. During the peripartum and lactation periods, AVP mRNA expression, receptor density, and binding are elevated (23). High maternal licking and grooming behavior correlates with increased V1aR density in the lateral septum of female mice (24). Infusion of a V1aR antagonist into the medial preoptic area (MPOA) delays the onset of maternal care (25), highlighting the importance of this receptor, although early interpretations attributed this to cross-talk of AVP to OT receptors. More recent studies support a distinct, OT-independent role for AVP in maternal care (4) and maternal aggression (26) in rats. This role has been replicated in other rodent species (27, 28) and in mammals more broadly (29, 30). Unlike OT, local AVP release into the MPOA and bed nucleus of the stria terminalis (BNST) increases during the display of maternal care and mother-pup interactions in rats (31). Although AVP levels in the BNST may not sharply increase with maternal behavior, the region houses AVP-synthesizing neurons and V1aR, which are upregulated during lactation (32, 33). While V1aR signaling in the BNST does not seem essential for ongoing pup retrieval and nursing, it is implicated in maternal aggression, which serves to protect the offspring (34). Elevated V1aR mRNA expression has been reported in the amygdala, SON, and lateral septum of postpartum multiparous rats compared to primiparous controls (23, 35). Maternal aggression, but not maternal care, is reduced following microinjection of a V1aR antagonist into the BNST of Brattleboro rats, suggesting region-specific regulation of maternal behaviors (36). Fluctuations in V1aR in several important regions of the central nervous systems appear to modulate maternal aggression during the peripartum period (37). Collectively, these findings suggest that V1aR contributes to maternal caregiving and aggression across rodent models.

Here, we investigated female sexual behavior and early postpartum maternal care in Syrian hamsters lacking V1aR, generated using CRISPR-Cas9 gene editing (38). Syrian hamsters have been extensively used in studies of social neuroscience because of their well-described patterns of social behaviors, which are strongly regulated by AVP (39). Syrian hamsters provide a valuable model because their maternal care is brief and energetically constrained compared to that of mice and rats, and maternal cannibalism is relatively common (40, 41). Unlike other rodents, maternal care in hamsters is often brief and energetically constrained, suggesting specialized neuroendocrine regulation. We previously observed altered aggression and social communication in V1aRKO hamsters of both sexes (38). Based on prior findings linking V1aR to maternal care and sociosexual signaling, we hypothesized that V1aR deletion would selectively impair sexual receptivity and maternal caregiving. By testing both copulatory interactions and pup-directed behaviors in V1aR KO, heterozygous (Het), and wild-type (WT) females, this study expands our understanding of the role of AVP in female reproductive behavior and highlights the species-specific contributions of V1aR to maternal investment.

## Materials and methods

### Subjects

Knockout Syrian hamsters (*Mesocricetus auratus*) were generated as previously described (38). A single founder female was bred to produce two litters by WT sires. Offspring were outbred to WTs for two more generations. Male and female Hets from F3 were crossed to produce WTs, Hets, and KOs. All subjects used for data collection were from F3 or their descendants. WTs, which were generated from Het × Het or Het × WT crossings, were used whenever possible, but some WTs were from WT litters whereas others purchased from Charles River Laboratories (CRL). The CRISPR-Cas9 strategy used in this study generated a germline (global) deletion of the Avpr1a gene, resulting in loss of V1aR expression in both central and peripheral tissues (38). All female subjects were virgin at the time of testing and around 60 days old. All females were primiparous and single-housed for at least 14 days prior to behavioral testing. Estrous cycles were monitored daily via vaginal discharge (42). Weanlings were kept in same-sex groups of three to six until adulthood. All hamsters were genotyped using samples from ear clippings (Transnetyx) and the following 4 groups were used in the study: control female Syrian hamsters purchased from Charles River Laboratories (CRL), WT, Het, and KO hamsters produced by our colony. Although, no significant differences emerged between CRL WT and colony WT females, we analyzed these groups separately to check for colony-specific effects. All males used in sexual behavior tests were CRL WT animals.

Syrian hamsters were single-housed in a 14-h light/10-h dark cycle (lights off at 10am), and provided food and water ad libitum. Previous studies demonstrated that single housing is not stressful for male and female Syrian hamsters (43). All procedures were approved by Georgia State University’s Institutional Animal Care and Use Committee and conformed to the NIH Guide for the Care and Use of Laboratory Animals (44), as well as the Animal Welfare Act Code of Federal Regulations. The Georgia State University animal care and use program is fully accredited by AAALAC International.

### Behavioral analysis

For all behavioral tests, videos were recorded from the side of the cage and later scored using The Observer (Noldus Information Technology B.V., Leesburg, VA) by 2 observers blind to the hamsters’ genotype. Inter-rater reliability (i.e., percent agreement) between the two observers was above 90%. Some behaviors (e.g., stereotypies, infanticide) were scored but too rare for statistical analysis and not shown in the results. Sexual behavior was recorded during the dark phase of the light cycle, approximately 1 hour after lights off. Dim red light was used for these recordings, as well as for video-recording maternal behavior during the dark phases.

### Sexual behavior

Female subjects were tested for copulatory behavior when paired with a sexually experienced male hamster from Charles River Laboratories. Females were tested in their home cage during the estrus phase of their estrous cycle. Copulatory tests lasted 30 minutes or until females began displaying aggression toward the male.

For females, duration, latency and number of lordosis responses were recorded. To evaluate whether the genotype of the female was able to induce differences in the mating behavior of the male hamsters, the duration, latency and number of the following male behaviors were scored: mounts, ectopic mounts, thrusting, anogenital investigation, self-grooming, social behaviors (stretch, approach, sniff, nose touching, and flank marking) and non-social behaviors (locomotion, exploration, feeding, and sleeping). Latency to first intromission was also recorded to evaluate sexual drive in males. Mounts and thrusting were distinguished because long intromissions are characterized by a repetitive thrusting pattern that differs from regular intromission, after which the male quickly dismounts the female following vaginal penetration. The expression of thrusting and long intromissions is associated with the onset of sexual satiety in this species (45, 46).

Other parameters related to pregnancy were not affected by genotype. Almost all copulatory tests resulted in successful pregnancy. Only one WT female hamster resulted in no pregnancy after copulation. No differences among groups were observed in the number or sex ratio of pups at weaning (PND 21; **Supplementary Figure 1**). The number of pups at birth was not collected to avoid disturbing maternal care and interfering with the rest of our study.

### Maternal behavior

Maternal behavior was continuously recorded for the first 4 days after delivery. Dams’ behavior was scored for 5-minute intervals four times per day: two times in the light phase (6:00am and 10:00pm) and two times in the dark phase (10:00am and 6:00pm). Thus, maternal behavior was scored at zeitgeber times (ZT) 20, ZT 0, ZT 8, and ZT 12. Both the position in relation to the nest as well as maternal and non-maternal behaviors were scored. **Table 1** summarizes the behaviors scored.

**Table 1:** List of behaviors scored for maternal behavior.

Ethogram maternal behavior	
Behavior	Description
Nest Position	
Crouching	Crouching over pups and immobile. Presumably nursing
Hovering	Hovering over nest and active
In nest	In nest, but not actively taking care of pups
Off nest	Dam off nest
Pup off nest	Pup/s off nest
Maternal Behavior	
Licking/Sniffing	Licking or sniffing the pups
Nesting	Building nest or related behaviors
Infanticide	Killing or eating pups
Pup retrieval	Pup retrieval
Other Behaviors	
Eating	Active eating chow
Drinking	Active drinking
Self-grooming	Self-groom and scratch
Locomotor Horizontal	Includes walk, explore, sniff that does not fall into any of the above categories
Active Vertical	Rear and lean on wall, lid sniff, lid chew (two paws on the floor of the cage), single jump
Bury	Moving of bedding forwards with forepaws
Dig	Moving of bedding backwards with forepaws
Inactive	Sit, laydown, sleep, freeze
Stereotypies	Spin turns, repeated jumps, repeated lid chews (>3), head shakes, etc.

Because no differences were observed between data collected during the light and dark phases, the results were collapsed over time and averaged, resulting in a single value of each behavior per day. Lastly, behaviors were grouped to create indices that reflect major behavioral components. **Table 2** shows the list of grouped behaviors and the single behaviors forming them.

**Table 2:** List of grouped and composite behaviors.

Grouped behaviors	List of behaviors
Maternal care	Licking/sniffing pups, pup retrieval, nesting behavior, hovering, crouching, time in nest
Total activity not in nest	Locomotor horizontal, locomotor vertical, self-grooming, eating, drinking, digging, and burying (when performed off nest)
Total locomotor activity	Locomotor horizontal, and locomotor vertical
Feeding behavior	Eating, and drinking
Burrowing	Digging, and burying

### Statistical analysis

Data were analyzed using Prism 9 (GraphPad), utilizing between subject analysis of variance (ANOVA). In cases of statistical significance, Tukey’s *post-hoc* tests were used to examine group differences (p < 0.05). For maternal behavior, scored behaviors were analyzed using a one-way ANOVA followed by Tukey’s *post hoc* tests was performed to test differences between groups per PPD. If data failed the assumption of the normal distribution of the residual, the non-parametric Kruskal-Wallis test was performed, followed by a Dunn-Bonferroni pairwise comparison using a significance value adjusted by the Bonferroni correction for multiple tests (adj. p < 0.05). For brevity, non-significant results are not reported unless meaningful.

## Results

### V1aR deletion fails to affect lordosis but reduces the number of mounting attempts received by female hamsters

**Figure 1:**
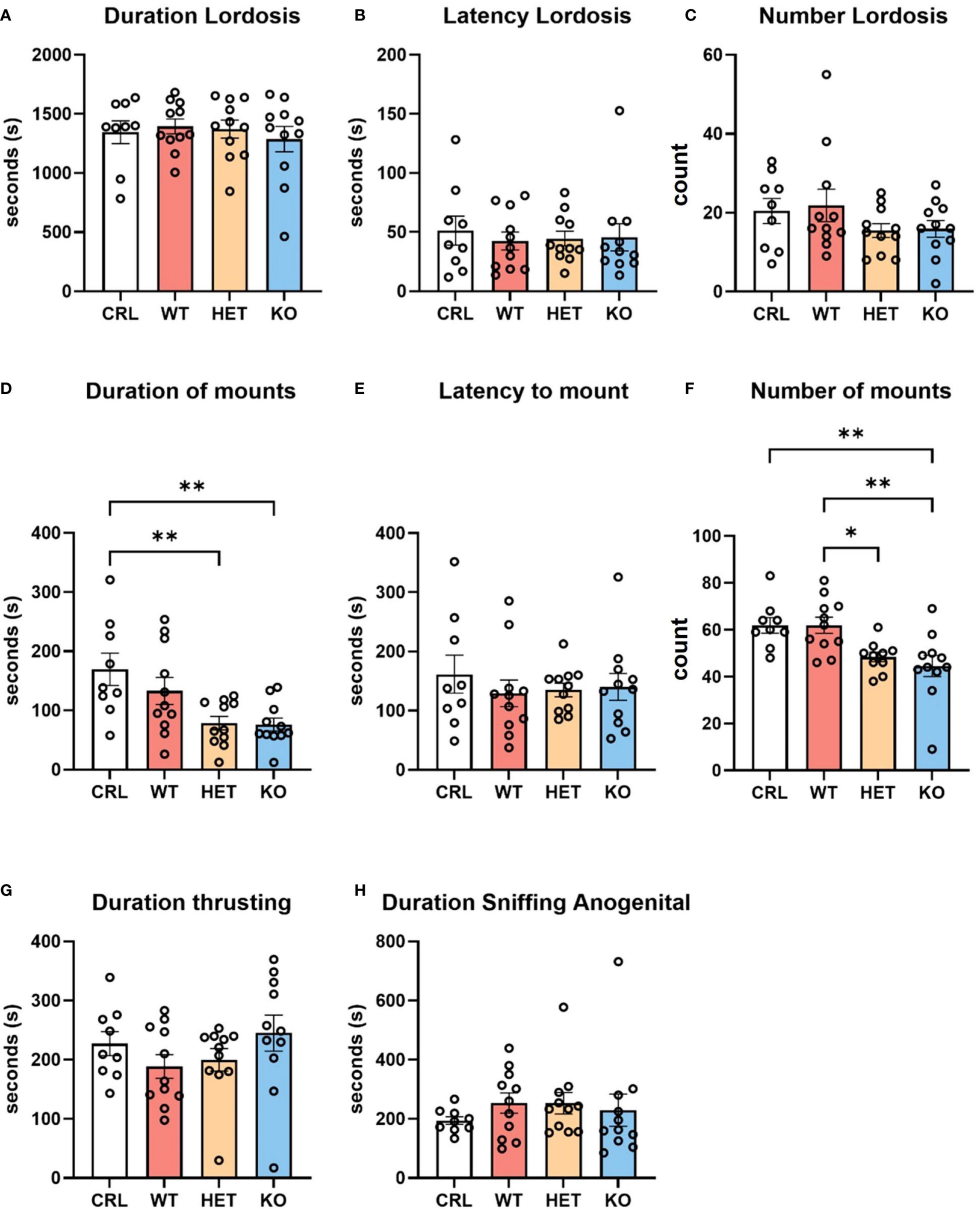
Effects of genotype on sexual behavior. The genotype of the females did not influence the duration **(A)**, latency **(B)**, or number **(C)** of lordosis episodes. However, male behavior was affected by the genotype of the female. Male hamsters showed longer duration **(D)** and higher frequency **(F)** of mounting attempts with CRL and WT females than with Het or KO females. Other male behaviors, such as latency to mount **(E)**, duration of thrusting **(G)** and anogenital sniffing **(H)** were not affected by female genotype. Bars represent the mean, error bars represent SEM, and the open circles represent one or more data points. * p<.05, ** p<0.01.

The genotype of the females did not significantly influence sexually receptive behavior in females (**Figure 1**). One-way ANOVA did not reveal any significant effects of genotype on lordosis duration (F (_3, 38_)=0.2908, p=0.83, η^2^ = 0.02; **Figure 1A**), lordosis latency (F (_3, 38_)=0.1409, p=0.93, η^2^ = 0.01; **Figure 1B**) or lordosis frequency (F_(3, 38)_=1.229, p=0.31, η^2^ = 0.08; **Figure 1C**).

Statistical analysis did reveal, however, an effect of female genotype on the behavior emitted by stimulus males. Since no aggression was observed towards males, these changes in male behavior suggest that the deletion of V1aR may affect signaling cues during mating. Specifically, one-way ANOVA showed an effect of genotype on the duration (F_(3, 38)_=5.565, p<0.01, η^2^ = 0.30; **Figure 1D**) and number (F_(3, 37)_=6.652, p<0.01, η^2^ = 0.35; **Figure 1E**) of male mounting attempts. Tukey’s HSD *post hoc* analysis indicated a significantly greater mounting duration by males in the CRL group compared to Het (p<0.01) and KO (p<0.01) groups. Likewise, males displayed significantly fewer mounting attempts with KO females than with CRL (p<0.01) and WT (p<0.01) females. Males also showed significantly more mounting attempts with WT than Het females (p<0.05). No other mating behaviors were significantly affected by female genotype, including duration of thrusting (**Figure 1G**) and duration of anogenital sniffing (**Figure 1H**).

### The complete deletion of V1aRs reduced the expression of maternal behavior

Statistical analyses revealed multiple significant behavioral differences across genotypes, with KO females displaying reduced maternal care and increased non-maternal behaviors.

**Figure 2:**
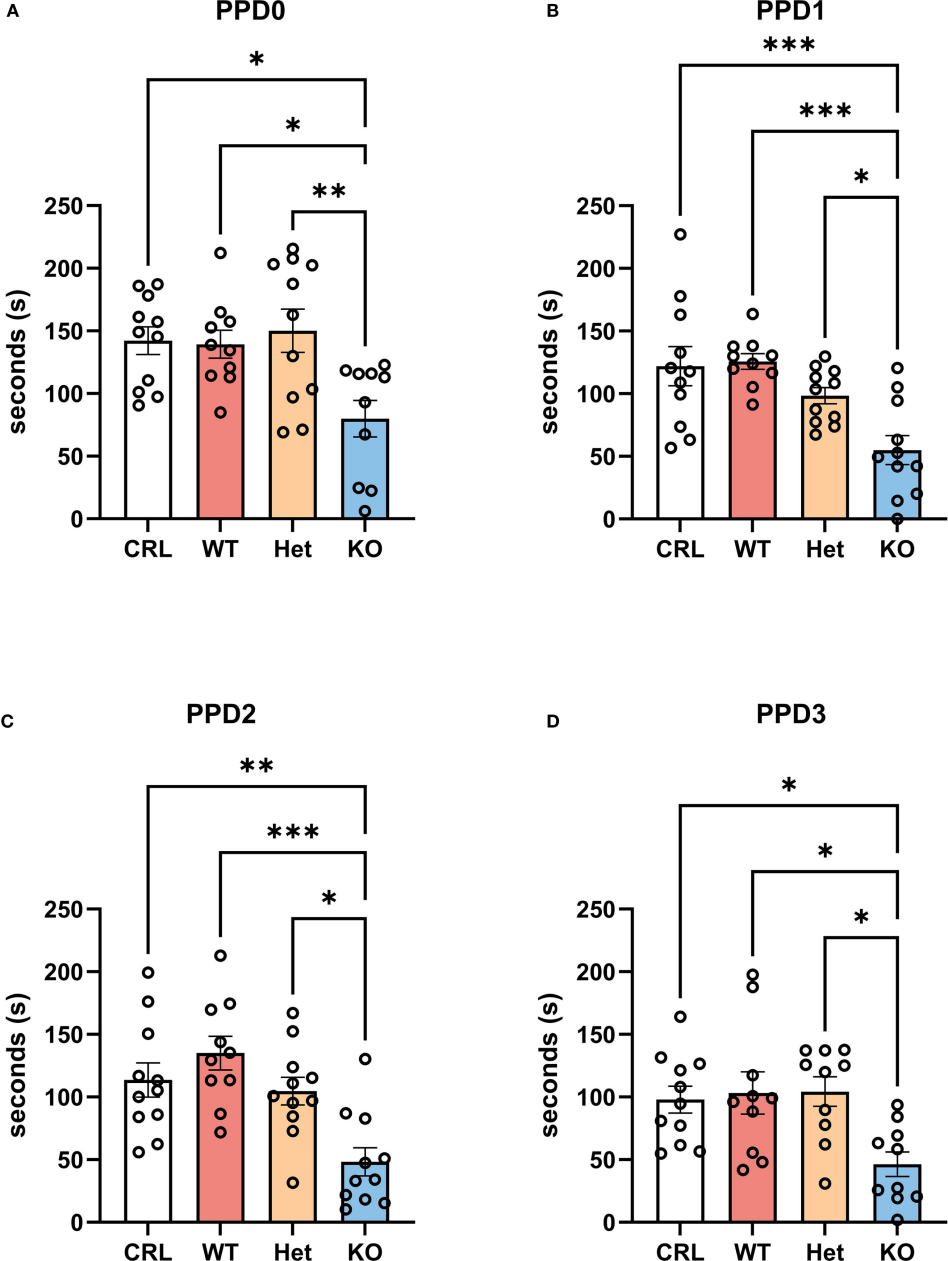
The deletion of V1aRs reduced maternal care in the first 3 days postpartum. V1aRKO female Syrian hamsters exhibited less maternal care across postpartum days (PPDs) and compared to all other genotypes. **(A)** shows the duration of maternal care from PPD0 to PPD3 for all four groups, indicating a reduction of maternal care during the days after parturition. Further, the statistical analysis revealed less maternal care in the KO group in comparison to CRL and WT hamsters on PPD0 **(B)**, PPD1 **(C)**, PPD2 **(D)**, and PPD3 (E.). Bars represent the mean, error bars represent SEM, and the open circles represent one or more data points. * p<.05, ** p<0.01, ** p<.001.

For maternal care, one-way ANOVA showed a genotype effect on PPD0 (F_(3, 38)_=5.28, p<0.01, η^2^ = 0.29; **Figure 2A**), PPD1 (F_(3, 39)_=8.97, p<0.001, η^2^ = 0.41; **Figure 2B**), PPD2 (F_(3, 39)_=8.89, p<0.001, η^2^ = 0.41; **Figure 2C**), and PPD3 (F_(3, 37)_=4.85, p<0.01, η^2^ = 0.28; **Figure 2D**). On PPD0, the *post hoc* tests revealed that KO dams exhibited significantly less maternal care than did CRL (p<0.05), WT (p<0.05) and Het (p<0.01) dams. Similar effects were highlighted by the Tukey’s *post hoc* analysis on the other PPDs with a significant reduction in the duration of maternal care emitted by the KO group in comparison to CRL (p<0.001, PPD1; p<0.01, PPD2; p<0.05, PPD3), WT (p<0.001, PPD1; p<0.001, PPD2; p<0.05, PPD3), and Het (p<0.05, PPD1; p<0.05, PPD2; p<0.05, PPD3) females.

**Figure 3:**
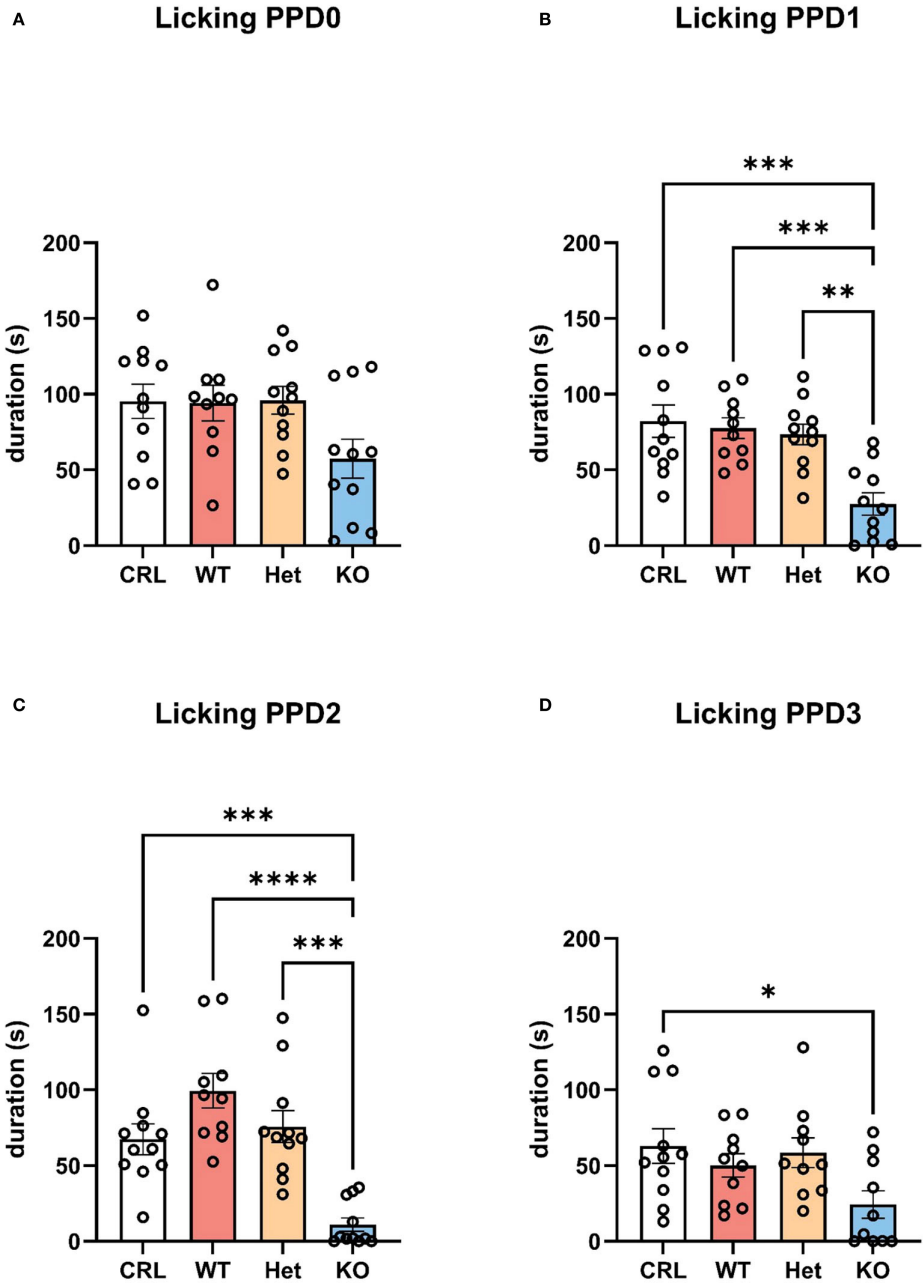
V1aR deletion specifically reduced active maternal care in the first 3 days postpartum. V1aRKO female Syrian hamsters exhibited less licking and grooming behaviors towards the pups compared to all other genotypes. The bar graphs show a trend of reduced duration of licking/grooming in KO animals compared to other genotypes on PPD0 **(A)**, which become significant on PPD1 **(B)**, PPD2 **(C)**, and PPD3 **(D)**. Bars represent the mean, error bars represent SEM, and the open circles represent individual data points. * p<.05, ** p<0.01, ** p<.001, ****p<.0001.

By analyzing the single behaviors composing maternal care, the statistical analysis indicated that licking/grooming the pups (*active maternal caregiving*) was the main behavior driving the observed differences among groups (**Figures 3A–D**). The one-way ANOVA revealed a significant effect of genotype on PPD1 (F_(3, 42)_=9.793, p<0.001, η^2^ = 0.43; **Figure 3B**), PPD2 (F_(3, 42)_=15.460, p<0.001, η^2^ = 0.54; **Figure 3C**), and PPD3 (F_(3, 42)_=3.142, p<0.05, η^2^ = 0.20; **Figure 3D**). Tukey’s HSD test indicated that KO hamsters licked/groomed less their pups than CRL (p<0.001), WT (p<0.001), and Het (p<0.01) dams on PPD1. The same statistically significant difference was reported by the *post hoc* analysis on PPD2 with less licking/grooming emitted by KO dams than CRL (p<0.001), WT (p<0.001), and Het (p<0.001) females. On PPD3, only CRL animals showed a significant higher duration of licking/grooming in comparison to KO dams (p<0.05).

**Figure 4:**
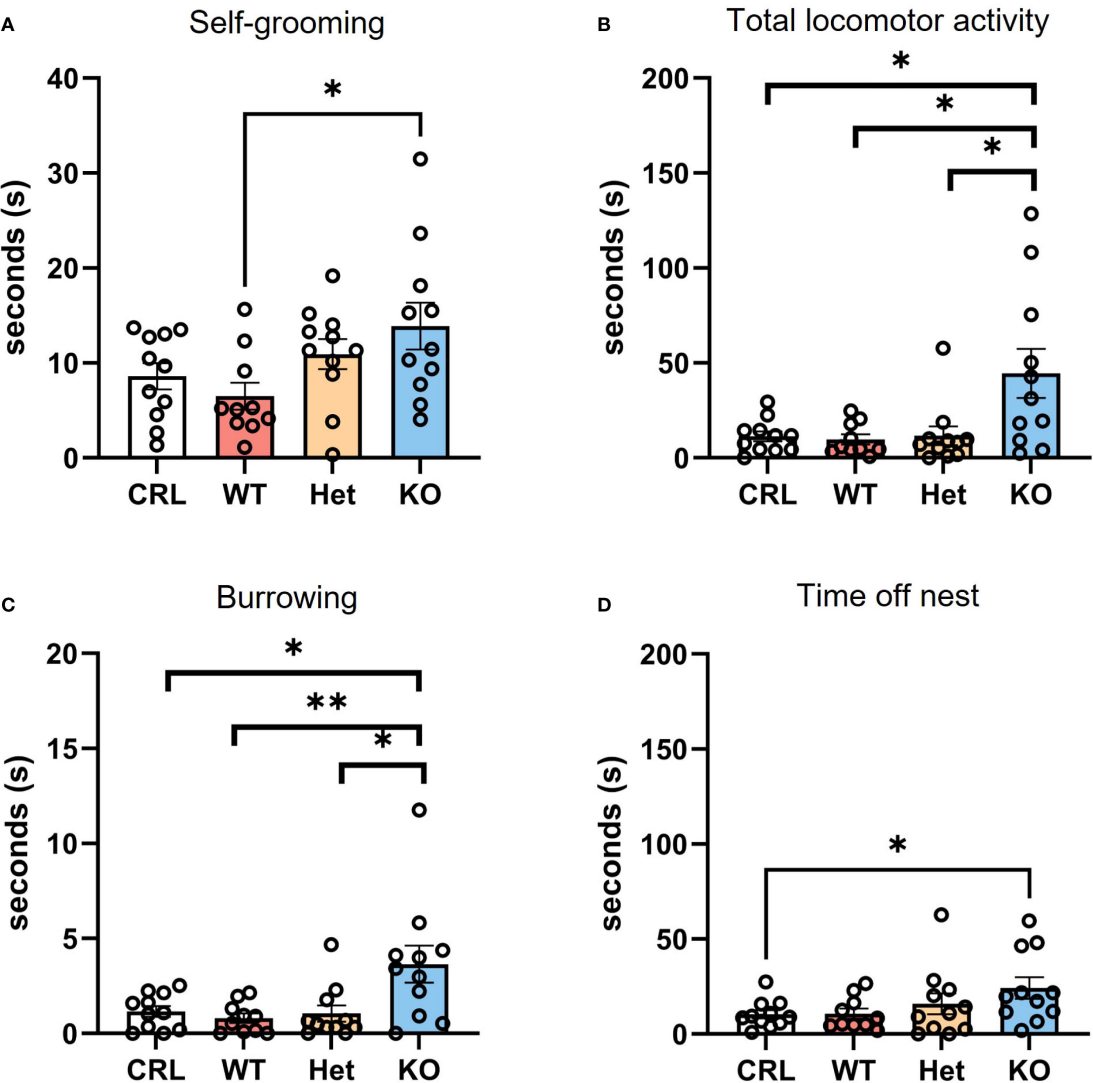
V1aR deletion alters postpartum behavior in dams in the postpartum period. During the first four days of postpartum, the genotype of hamsters deeply impacted the behaviors emitted by dams. Self-grooming was significantly higher in KO animals compared to WT on average **(A)**. V1aRKO females also displayed a statistically significant higher locomotor activity **(B)** and burrowing behavior **(C)** in comparison to all other groups. In addition, the time spent off the nest was also more in the KO group than CRL dams **(D)**. Bars represent the mean, error bars represent SEM, and the open circles represent one or more data points. * p<.05, ** p<0.01.

Other behaviors were also affected by genotype. Since observations of these behaviors were less frequent, we decided to present the data as average of duration across the four different PPD. For self-grooming, the Mann-Whitney test revealed significantly higher average self-grooming in the KO group compared to WT (U = 21, z=-2.495, p<0.05; **Figure 4A**). For total locomotor activity, the Mann-Whitney test indicated a significant increase in total locomotor activity in the KO females than CRL (U = 29, z=-1.183, p<0.05; **Figure 4B**), WT (U = 2513.5, z=-2.247, p<0.05; **Figure 4B**), and Het (U = 26, z=-2.386, p<0.05; **Figure 4B**). The Kruskal-Wallis test identified an effect of genotype on burrowing behavior (digging and burying; H_(3)_=9.50, p<0.05, η^2^ = 0.29; **Figure 4C**). The Mann-Whitney analysis indicated more burrowing in KO hamsters in comparison to CRL (U = 26, z=-2.025, p<0.05) WT (U = 18, z=-2.272, p<0.01) and Het (U = 25, z=-2.552, p<0.05) groups. The genotype of hamsters also affected the average amount of time spent off-nest (**Figure 4D**). In particular, the Mann-Whitney *post hoc* analysis showed that V1aRKO females spent significantly more time away from the nest than CRL (U = 28, z=-1.851, p<0.05).

## Discussion

In this study, we tested the hypothesis that V1aRs contribute to the regulation of sexual and maternal behavior. Although our results did not show an effect of V1aR deletion on the overall duration of lordosis behavior in female hamsters, the genotype of the female hamster significantly influenced male mating behavior, with males displaying fewer and shorter mounting attempts when paired with females lacking one or both copies of the *Avpr1a* gene. Interestingly, no differences were observed in male thrusting or anogenital sniffing, which may explain why copulation itself was unaffected by genotype, as evidenced by similar pregnancy success rates and pup numbers at weaning (**Supplementary Figure 1**). In addition to the effects on sexual behavior, V1aR deletion significantly altered maternal behavior, with KO females showing an overall reduction in pup-directed behavior and increased non-maternal behaviors. Specifically, AVP seems to modulate active maternal caregiving behaviors like licking and grooming the pups, as shown by our results. Despite spending similar amounts of time in the nest, V1aRKO females engaged less in maternal care, suggesting a selective caregiving deficit rather than general disinterest. Across the early postpartum period, KO females exhibited significantly increased locomotor activity, burrowing, and time off the nest compared with WT and Het females, whereas Het females showed a modest increase in self-grooming, the functional significance of which remains unclear. The increased burrowing behavior of V1aRKO females may reflect enhanced nesting activity; however, when considered alongside the increased locomotor activity, it could also indicate a form of anxiety-like behavior (47). Further analysis is needed to clarify the meaning of these behavioral changes. It is important to note that our CRISPR-Cas9 knockout strategy deletes Avpr1a globally, meaning that V1aR is absent not only in the brain but also in peripheral tissues such as vasculature and kidney, where it normally contributes to cardiovascular regulation, fluid balance, and stress responses (9). While we did not observe overt peripheral deficits, future work should address whether peripheral physiological consequences of V1aR loss emerge under stress or challenge conditions. Given our focus on maternal and sexual behavior, the most parsimonious explanation for our findings is that they arise from loss of V1aR signaling within brain regions critical for sociosexual behavior.

In female hamsters, sexual receptivity is primarily expressed via vaginal marking and lordosis, although these behaviors are not always co-expressed (48–50). Lordosis, in particular, facilitates intromission by arching the back and elevating the hindquarters and perhaps also blocking aggressive behavior in female hamsters, which are notoriously aggressive when not in estrus (51–53). Although we found no effect of V1aR deletion on overall lordosis duration, several studies support a role for AVP, and specifically V1aR, in this behavior. For instance, infusion of a V1aR antagonist in ventral pallidum of estradiol-primed female rats increases lordosis frequency, suggesting that AVP may have an inhibitory role (54). In agreement with this finding, systemic AVP administration also reduces lordosis (15), suggesting that AVP may act as a brake on female sexual behavior. The reduced lordosis after AVP administration may be due to the interaction with OT, as OT administration can reverse this effect (15). Conversely, exposure to short photoperiods (<12.5h of light/day), which induce anestrus, is associated with reduced V1aR binding in many brain regions important in sociosexual behavior (21), indicating complex interactions between photoperiod, AVP signaling, and sexual receptivity. The absence of lordosis differences in our V1aRKO groups may reflect compensatory mechanisms by other neuroendocrine systems, such as OT, during development.

An unexpected finding in our study was that V1aRKO females retained normal fertility: they conceived at similar rates to WT females, and produced comparable litter sizes and sex ratios. This outcome is intriguing in light of work showing that AVP, acting through V1aR, contributes to regulation of the preovulatory luteinizing hormone (LH) surge in female mice (55). Our results suggest that in Syrian hamsters, the LH surge and ovulation can proceed in the absence of V1aR signaling, perhaps through compensatory activation of oxytocin or V1b receptors, or species-specific differences in hypothalamic regulation of gonadotropin release. Thus, while V1aR is clearly important for aspects of sexual signaling and maternal care, its role in the neuroendocrine control of ovulation may be redundant or less critical in this species. Future studies that directly measure LH release in V1aRKO females will be necessary to clarify the contribution of AVP signaling to fertility.

Intriguingly, males showed a specific reduction in mounting behavior when paired with Het or KO females. No changes were observed in other behaviors such as thrusting or anogenital sniffing, which have been associated with sexual satiety and individual recognition, respectively (50, 56). The specific reduction in mounting behavior, therefore, suggests that males may be able to detect subtle differences in female cues that do not disrupt consummatory behavior once interaction begins. In rodents, males rely heavily on olfactory signals, both volatile odors and non-volatile chemosignals, to assess female receptivity. V1aR may influence the composition or detectability of these signals, perhaps through altered neuroendocrine pathways. Prior research shows that V1aR knockout or antagonism reduces pre-copulatory behavior and alters social signaling, including scent marking (21, 38). Additionally, V1aR may affect female behavioral cues such as approach or pacing, which in turn influence male mounting behavior. Even when lordosis behavior is preserved, these subtler social cues can strongly impact male mounting behavior. Brain regions such as the lateral septum, where V1aR is expressed, are known to modulate sensory detection and social responses (57). Notably, we observed that Het and KO females frequently moved away immediately after the male ceased investigating or mounting, which may account for the observed reduction in mounting behavior and is consistent with the higher locomotion duration in the KO group. However, the high variability in male and female interactions (e.g., males often exhibit intermittent mounting or cease mating at different times, and some females display early aggression that prevents mounting) made systematic quantification difficult; future studies may consider alternative approaches to capture these subtle behavioral differences. While the role of AVP in female sexual behavior remains incompletely understood, our findings suggest that AVP may influence female sexual signaling in ways that indirectly affect male behavior. Alternatively, AVP may contribute to the maintenance or consistency of lordosis posture, and its absence could subtly disrupt behavioral feedback loops during courtship and mating.

Our results also show that V1aR deletion significantly reduces maternal behavior while sparing other behaviors, aligning with existing literature. In rodents, AVP acting via V1aR has been shown to facilitate maternal responsiveness, particularly in regions such as the MPOA and the BNST, both critical hubs for maternal behavior (2, 58, 59). Further, V1aR expression correlates with licking/grooming and nursing behaviors, with increased expression correlated to naturally high licking and grooming dams (60). In agreement with this, V1aR antagonism or knockdown leads to decreased nurturing behaviors in lactating rats (2). Additionally, reduced V1aR binding in the MPOA has been linked to lower levels of maternal-pup contact and fewer nurturing postures (2). Although most prior work has focused on mice and rats, similar patterns emerge across species. In prairie voles, for example, central V1aR activation promotes pup retrieval and nesting, while V1aR disruption impairs maternal responses (61). Interestingly, in our study, V1aRKO females showed increased burrowing and locomotor activity, which may reflect a shift in behavioral priorities or disinhibition of exploratory drive. AVP is implicated in arousal regulation and behavioral gating (62), so V1aR loss may dysregulate these processes. Importantly, the increase in non-maternal activity co-occurred with decreased maternal behavior, indicating that V1aRKO females shifted their behavioral output away from pup-directed care toward other activities, despite spending similar amounts of time in the nest. This distinction supports the idea that V1aR regulates affiliative behaviors independently of overall motor activity (2). Together, these results suggest that V1aR is a critical regulator of maternal care in Syrian hamsters, likely acting through conserved neural circuits that govern social and parental behaviors across species. Our data further highlight the behavioral selectivity of V1aR signaling, which may offer insights into the nuanced neuropeptide regulation of parental investment and the evolution of care-giving strategies in mammals.

In summary, our findings demonstrate that V1aR knockout in female Syrian hamsters selectively alters key facets of social and reproductive behavior, without broadly impairing sociosexual behavior. Copulatory behavior and nest occupancy were preserved, yet KO females showed reductions in subtle aspects of sexual signaling, reflected in decreased male mounting responses, and exhibited markedly less active maternal care, particularly pup licking and grooming. At the same time, V1aRKO females displayed elevated non-maternal behaviors such as burrowing and self-grooming, suggesting a shift in behavioral priorities rather than a simple loss of maternal responsiveness. Taken together, these findings indicate that V1aR contributes selectively to certain components of sexual communication and maternal caregiving, while also modulating the balance between caregiving and other behaviors. Future studies should examine how V1aR deletion shapes the underlying neural circuits governing this behavioral allocation, as well as their involvement in human disorders characterized by social and maternal behaviors, such as postpartum depression. A deeper understanding of V1aR’s role in affiliative behavior could inform both evolutionary models and translational treatments targeting social behavior.

## Data Availability

The raw data supporting the conclusions of this article will be made available by the authors, without undue reservation.

## Supplementary material

Supplementary Figure 1 Effects of genotype on total number and sex ratio of pups at weaning PND21. The genotype of the females did not affect the total number **(a)** of pups or their sex ration **(b)** at weaning (PND21). Bars represent the average number of pups and the open circles represent individual data points.
