# Supplementary figures and images for: The deletion of the arginine vasopressin 1a receptor impairs sexual and maternal behavior
Source: Front Endocrinol (Lausanne). 2025 Sep 17;16:1649706. doi: 10.3389/fendo.2025.1649706 (PMC12483901; doi:10.3389/fendo.2025.1649706)

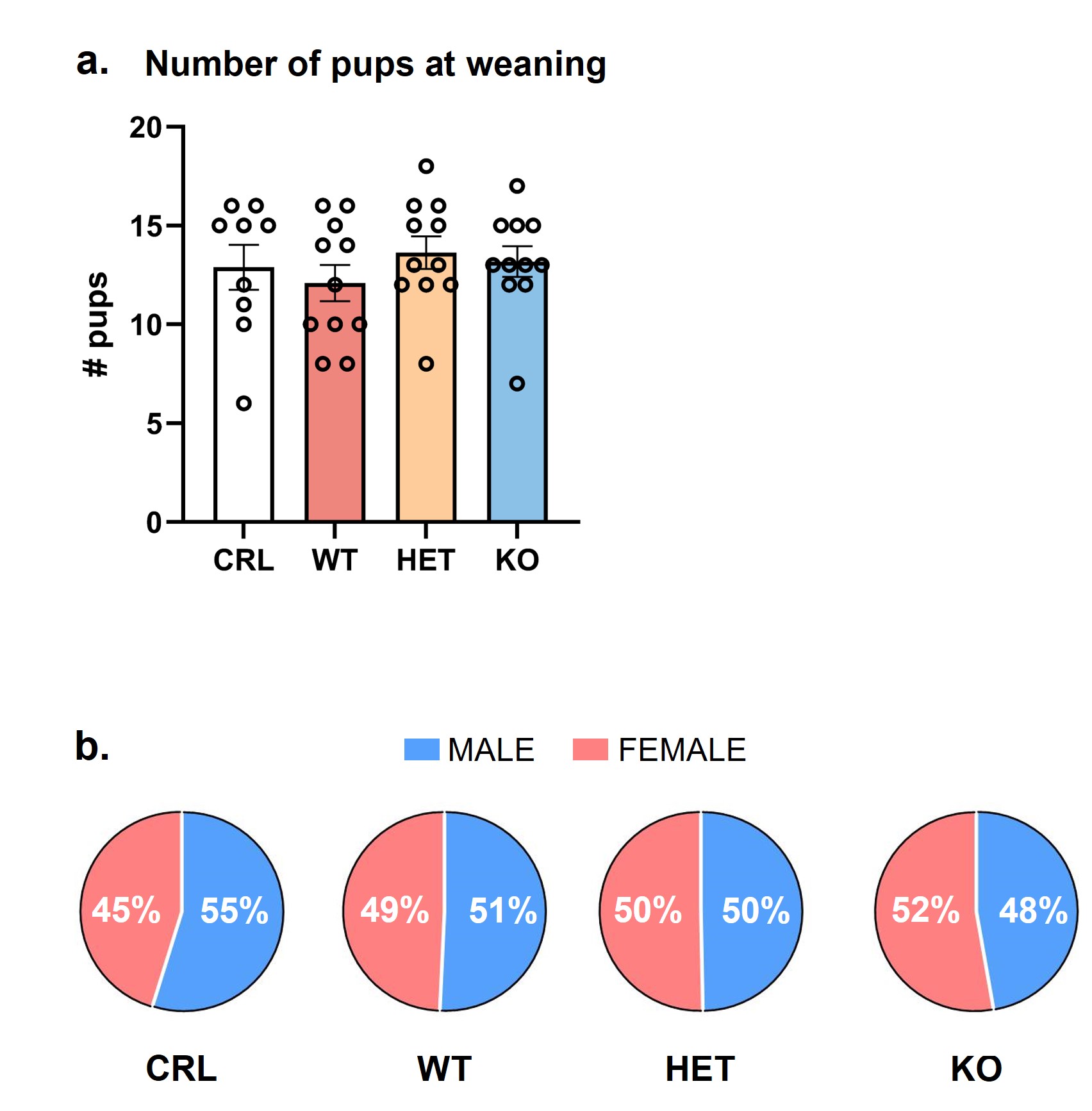

Supplement: Supplementary Figure 1 — Effects of genotype on total number and sex ratio of pups at weaning PND21. The genotype of the females did not affect the total number (a) of pups or their sex ration (b) at weaning (PND21). Bars represent the average number of pups and the open circles represent individual data points. [file Image1.jpeg]
